# In Vitro Assessment of Biocontrol Effects on Fusarium Head Blight and Deoxynivalenol (DON) Accumulation by DON-Degrading Bacteria

**DOI:** 10.3390/toxins12060399

**Published:** 2020-06-16

**Authors:** Hiroyuki Morimura, Michihiro Ito, Shigenobu Yoshida, Motoo Koitabashi, Seiya Tsushima, Maurizio Camagna, Sotaro Chiba, Daigo Takemoto, Kazuhito Kawakita, Ikuo Sato

**Affiliations:** 1 Graduate School of Bioagricultural Sciences, Nagoya University, Furo-cho, Chikusa-ku, Nagoya, Aichi 464-8601, Japan; morimura.hiroyuki@e.mbox.nagoya-u.ac.jp (H.M.); camagna@agr.nagoya-u.ac.jp (M.C.); chiba@agr.nagoya-u.ac.jp (S.C.); dtakemo@agr.nagoya-u.ac.jp (D.T.); kkawakit@agr.nagoya-u.ac.jp (K.K.); 2 Tropical Biosphere Research Center, University of the Ryukyus, 1 Senbaru, Nishihara-cho, Okinawa 903-0213, Japan; michihi@comb.u-ryukyu.ac.jp; 3 Central Region Agricultural Research Center, National Agriculture and Food Research Organization, 2-1-18 Kannondai, Tsukuba, Ibaraki 305-8666, Japan; yoshige@affrc.go.jp (S.Y.); koita@affrc.go.jp (M.K.); 4 Department of Molecular Biology, Tokyo University of Agriculture, 1-1-1 Sakuraga-oka, Setagaya-ku, Tokyo 156-8502, Japan; seiya.tsushima@gmail.com

**Keywords:** *Fusarium graminearum*, trichothecene, deoxynivalenol, mycotoxin degradation, phytotoxin, biocontrol, *Nocardioides*, *Devosia*

## Abstract

Fusarium head blight (FHB) of cereals is a severe disease caused by the *Fusarium graminearum* species complex. It leads to the accumulation of the mycotoxin deoxynivalenol (DON) in grains and other plant tissues and causes substantial economic losses throughout the world. DON is one of the most troublesome mycotoxins because it is a virulence factor to host plants, including wheat, and exhibits toxicity to plants and animals. To control both FHB and DON accumulation, a biological control approach using DON-degrading bacteria (DDBs) is promising. Here, we performed a disease control assay using an in vitro petri dish test composed of germinated wheat seeds inoculated with *F. graminearum* (Fg) and DDBs. Determination of both grown leaf lengths and hyphal lesion lengths as a measure of disease severity showed that the inoculation of seeds with the DDBs *Devosia* sp. strain NKJ1 and *Nocardioides* spp. strains SS3 or SS4 were protective against the leaf growth inhibition caused by Fg. Furthermore, it was as effective against DON accumulation. The inoculation with strains SS3 or SS4 also reduced the inhibitory effect on leaves treated with 10 µg mL^−1^ DON solution (without Fg). These results indicate that the DDBs partially suppress the disease by degrading DON.

## 1. Introduction

**Figure 1:**
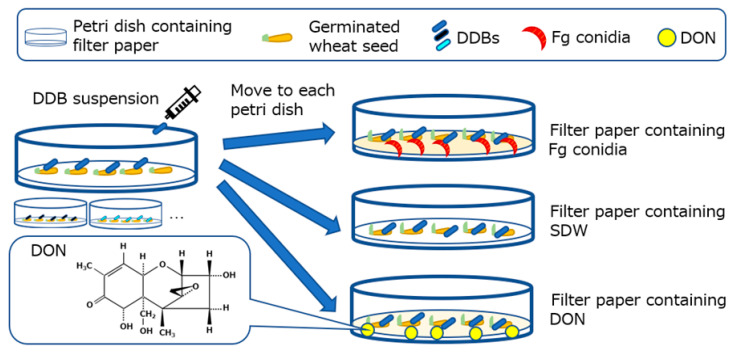
Overview of the experimental design used in this study. Structure of deoxynivalenol (DON: 3α, 7α, 15-trihydroxy-12, 13, epoxytrichotec-9-en-8-one) is shown on the lower left. The procedure of the petri dish tests: Wheat seeds were put on a filter paper inside a petri dish and germinated, DON-degrading bacteria (DDB) suspension was then dropped onto the germinated seeds (one suspension per petri dish). After one day of incubation, the germinated seeds were transferred to petri dishes containing filter papers treated with either *F. graminearum* s. str. 0407011 (Fg) (Figure 2), sterile distilled water (SDW) (Figure 3), or 10 µg mL^−1^ DON solution (Figure 4a,b). After an additional 6 days of incubation, the leaf and/or mycelia lengths were determined.

Fusarium head blight (FHB), caused by the *Fusarium graminearum* species complex, causes severe damage to wheat and barley throughout the world [1]. When the disease occurs in the spikelet, withering, and growth inhibition of cereals can lead to significantly reduced grain yield and quality. Moreover, it can contaminate grains with trichothecene mycotoxins [2,3,4,5,6]. The FHB occurrence is mainly reported in temperate areas such as East Asia, North America, and Europe, resulting in massive economic impacts. During the 1990s, the financial loss caused by FHB and mycotoxin, in the US alone, was estimated to be close to the US $3 billion [1]. The mycotoxins, which are produced by the *F. graminearum* species complex, include the trichothecenes, namely deoxynivalenol (3a,7a,15-trihydroxy-12,13-epoxytrichothec-9-en-8-one; DON; Figure 1), nivalenol (NIV) and T2 toxin, as well as fumonisin and zearalenone (ZEN). The mycotoxin contamination of food crops triggers above all, health problems. Notably, DON causes chronic toxicity such as immunosuppression, anorexia, and growth retardation, as well as acute poisoning such as diarrhea and vomiting [7,8]. DON is one of the most frequently detected mycotoxins in the food chain, and its concentration is commonly subject to regulation. In the case of Japan, the provisional concentration of DON in wheat grains was set at 1.1 mg kg^−1^ by the Ministry of Health, Labor, and Welfare in 2002. The joint WHO/FAO Expert Committee on Food Additives set a provisional maximum tolerable daily DON intake of 1 μg kg^−1^ body weight in 2001.

Chemical fungicides are used to prevent FHB and mycotoxin accumulation, but they cannot completely suppress the accumulation of DON [9]. According to Japanese regulations, chemical treatments are prohibited from two to three weeks before the harvest, despite the circumstance that both *F. graminearum* infection and DON accumulation could proceed during this period [6]. Moreover, in China, the resistance of *F. graminearum* to chemicals has been reported [10], indicating that continuous monitoring of DON accumulation is imperative to avoid contamination with this mycotoxin. In recent years, consumers have increasingly been expressing the desire for more secure and eco-friendly agricultural products. In order to respect the consumer’s needs, pest control techniques need to diversify and include novel environmentally friendly techniques. Eco-friendly biological control agents (BCAs) are an essential control technique in modern agriculture, and they have become an indispensable tool for approaches based on integrated pest management (IPM) [11]. Today, biocontrol microorganisms are successfully applied to fields in order to reduce crop damages caused by phytopathogens.

Several BCAs belonging to the genus *Bacillus* have been reported to control *F. graminearum* [12,13,14,15]. One article found, however, that the antifungal activity of *Bacillus* sp. led to an unintentional accumulation of DON [14]. Moreover, secondary metabolites, including antimicrobial agents, that were produced by BCAs could involve severe and unpredictable risks [16]. Thus, restrictive regulatory conditions for the application of BCAs were put in place in the EU [16].

DON acts as an essential virulence factor of the necrotrophic FHB pathogen in spreading the infection on wheat plants. During its initial infection, DON inhibits host programmed cell death (PCD). However, a high concentration of DON in the plant cell promotes the synthesis of H_2_O_2_, resulting in rapid apoptosis, and leads to inhibition of defense-related responses [17,18,19]. Previous studies have demonstrated that *tri5* knockout mutants, which are unable to produce DON due to their inability to convert the initial farnesyl pyrophosphate to trichodiene, are less virulent because the fungi could not spread into the rachis [20]. Since virulence factors are required in several pathogens to infect a host effectively, fungicides like mepanipyrim, which shows a remarkable potency against the phytopathogens *Botrytis cinerea* and *Sphaerotheca* sp., can prevent their infection owing to their ability to impede the extracellular secretion of the infectious agents [21]. Therefore, these findings indicate that suppression of DON accumulation in plants may represent a viable method for controlling the spread of *F. graminearum*.

The modes of action (MOAs) of how microbial BCA can suppress plant disease symptom can be divided into five main types as follows: (1) hyperparasitism/antibiosis; (2) competition with pathogens for nutrients and niche space; (3) induction of plant resistance against pathogens; (4) plant growth promotion [22,23]; and (5) degradation of pathogenesis-related compounds [24]. Several biocontrol agents are also considered to have multiple MOAs on disease suppression. Regarding the fifth MOA, degradation of pathogenesis related compounds can include various types of compounds, such as virulence factors/phytotoxins (e.g., fusaric acid produced by *F. oxysporum* [24]) as well as infection promoting factors (e.g., quormone produced by *Ralstnia solanacearum* [25]). DON-degrading bacteria (DDBs) would, therefore, be expected to act as a fifth MOA-type suppressor.

DDBs that have been isolated from various environments [26,27] appear to be promising BCAs against FHB and DON accumulation, although no reports employing disease control assays using plants have to our knowledge been published. Several studies on BCAs that have the ability to suppress both disease and phytopathogenic toxins have been documented. A non-phytopathogenic *Ralstonia solanacearum* strain, which can degrade fusaric acid (FA) produced by *F. oxysporum* f. sp. *lycopersici*, has been shown to stall disease progression [28]. Furthermore, the antagonistic effect of *Clonostachys rosea* strain IK726 against *F. graminearum*, which can degrade the mycotoxin ZEN, was also observed [29]. How the virulence of the FHB causing *F. graminearum* relates to DON-degradation is, however, so far an open question. A standard disease control assay for FHB is the quantification of the infection area of wheat spikes inoculated with *F. graminearum* in a greenhouse [30]. However, this method requires a great deal of labor, cost, time, and space and requires the growth stages of the wheat spike to be observed in detail [31]. To address these problems, Mesterházy [32] reported a petri dish test that assesses the incidence of the pathogen by the germination rate on wheat seeds. The results of this test were shown to highly correlate with the results obtained in the growth chamber and adequately reflect the pathogen incidence on a field scale [30]. Moreover, Purahong et al. [33] devised a modification to the in vitro test that calculates the petri dish aggressiveness index from the germination rate, coleoptile length reduction, and standardized area under the disease progress curve (AUDPC). Screening of BCA candidates is generally performed in a dual culture test on solid medium plates. This method cannot, however, be used to observe the adverse effects of the BCA on plants, which may be caused by mycotoxin accumulation. We have, therefore, devised a simple BCA screening assay using germinated wheat seeds, which addresses this problem (Figure 1). The method established in this study represents a promising new tool to efficiently screen for DDBs, which suppress both FHB and DON accumulation.

## 2. Results

### 2.1. Devosia sp. NKJ1 and Nocardioides spp. SS3 and SS4 Suppress the Disease Progression of Fg

**Figure 2:**
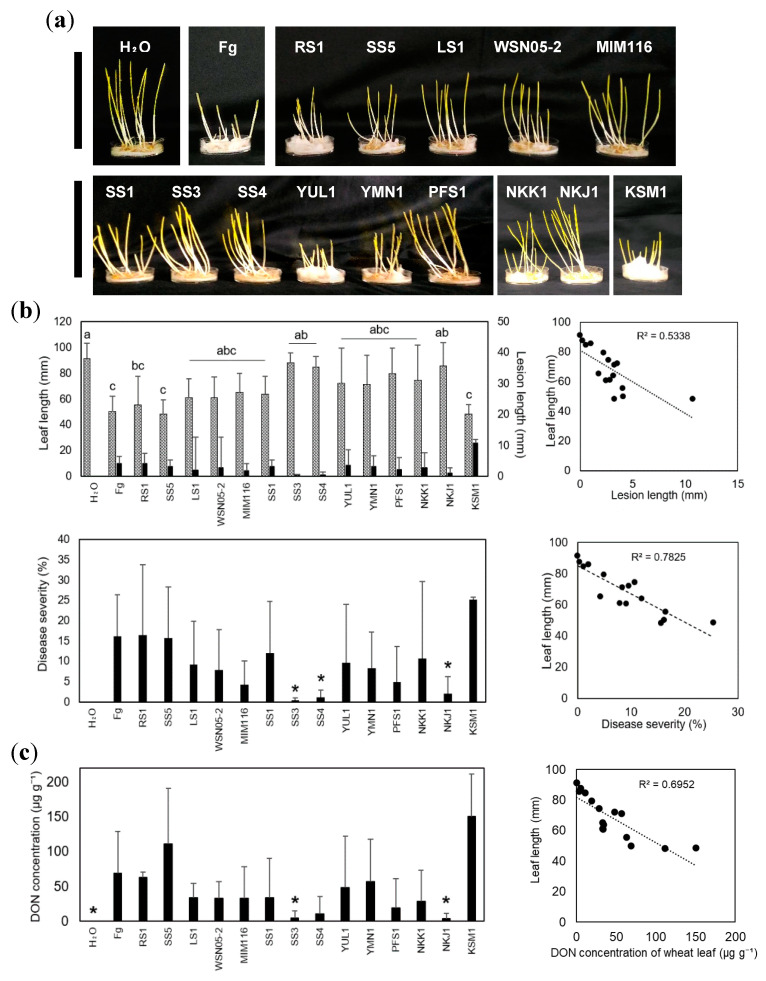
Suppression effects of DDBs on FHB and DON accumulation were examined using the petri dish test. (**a**) The pictures were taken at 6 days after inoculation. The black bars indicate 100 mm. (**b**) The upper left panel shows leaf lengths (dotted bars) and lesion lengths (closed bars) in the petri dish test. The lower left bar graph shows the disease severity in the trial. Fg indicates inoculation with *F. graminearum* s. str. 0407011 alone. H_2_O (sterile distilled water) means treatment without DON. The samples were inoculated with DON-degrading bacteria (DDBs). Each value shows the means of 9 repetitions for H_2_O and Fg and 6 repetitions for samples treated with DDBs. The error bars indicate standard deviation. The statistics analysis was performed by ANOVA, incorporating the Tukey–Kramer method. Samples marked by the same lower letters were not significantly different (*P* < 0.05). Asterisks (*), located on the disease severity, indicate significant differences by unpaired Student’s *t*-test between Fg and DDBs. The scatter diagrams to the right show the correlation between wheat leaf length and lesion length (upper panel) and between wheat leaf length and disease severity (lower panel). (**c**) Left panel, DON concentration of wheat leaves inoculated with Fg and DON-degrading bacteria. Error bars represent the standard deviation of the means of the results obtained with five replications. Statistical analysis was calculated using unpaired Welch’s *t*-test. Asterisks show significant differences (*P* < 0.05) between only Fg treatment and DON-degrading bacterial treatment. Right panel, the scatter plot indicates that a correlation was present between wheat leaf length and DON concentration of wheat leaf.

We used DDBs that had previously been isolated in Japan. Each DDB has different DON-degrading activity and a taxon-dependent DON metabolic pathway (Table 1) [34,35,36,37]. Germinated wheat seeds, challenged by *Fusarium graminearum* s. str. 0407011 (Fg) [38], were observed at 6 days after inoculation (DAI) with Fg in the petri dish test (Figure 2a,b). The wheat leaf lengths inoculated by Fg together with *Devosia* sp. NKJ1 (leaf length: 85.7 mm), *Nocardioides* sp. SS3 (leaf length: 87.7 mm) and SS4 (leaf length: 84.6 mm) were significantly longer than those which had only been inoculated with Fg (leaf length: 50.1 mm). In the inoculation of strain NKJ1(lesion length: 1.0 mm), SS3 (lesion length: 0.2 mm) and SS4 (lesion length: 0.6 mm), the lesion lengths were slightly shorter than only Fg inoculation (lesion length: 4 mm). Furthermore, a correlation existed between the wheat leaf lengths and the lesion lengths (Figure 2b, R^2^ = 0.53, P < 0.05). Comparison of the disease severities, calculated as the lesion length relative to the length of the whole leaf, demonstrated that the severities were significantly lower for the strains NKJ1 (severity: 2%), SS3 (severity: 0.3%), and SS4 (severity: 1.1%) when compared to wheat seeds inoculated with Fg alone (severity: 16.2%) (unpaired Student’s t-test, P < 0.05). A comparison of the disease severity and leaf length of the germinated seeds revealed a strong negative correlation (Figure 2b, R^2^ = 0.78, P < 0.05), whereby a higher disease severity coincided with a reduction in leaf length. The results indicated that *Devosia* sp. NKJ1 and *Nocardioides* spp. SS3 and SS4 suppressed the disease progression of Fg in germinated wheat seeds.

### 2.2. Devosia sp. NKJ1 and Nocardioides spp. SS3 and SS4 Prevent the DON Accumulation in Wheat Leaves

Measurement of the DON concentrations in germinated wheat leaves, co-incubated with Fg and DDBs, revealed that *Devosia* sp. NKJ1 and *Nocardioides* spp. SS3, which had indicated a suppressive suppression effect on disease progression, also showed a significant reduction in DON-accumulation when the low accumulation of DON was compared to the inoculations of Fg alone (Figure 2c). Besides, other DDB-treatments such as *Nocardioides* spp. SS4 may have led to a reduction of DON accumulation, although this reduction was not found to be statistically significant (P = 0.13). Comparative analysis between disease severity and DON accumulation showed highly significant correlations with R^2^ = 0.69 (*P* < 0.05) (Figure 2c).

### 2.3. Devosia sp. NKJ1 and Nocardioides spp. SS3 or SS4 Did Not Promote Wheat Growth

**Figure 3:**
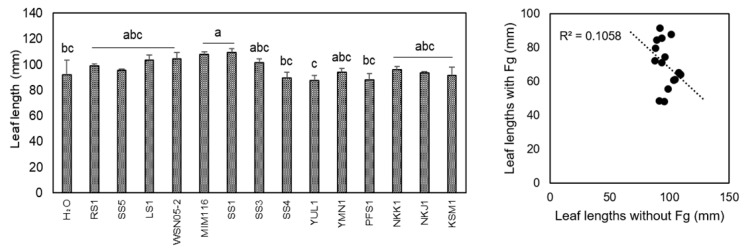
The leaf lengths of the germinated wheat seeds inoculated with DBBs. The experiment used the same petri dish test method, but without inoculation of *F. graminearum* s. str. 0407011 (Fg). H₂O (sterile distilled water) indicates a treatment without DDBs. The error bars indicate standard deviation. Data are the mean of triplicates. The statistics analysis was performed by ANOVA, incorporating the Tukey–Kramer method. The samples marked by the same lower letters were not significantly different (*P* < 0.05).

In order to survey whether DDBs exert an influence on wheat leaf length, the petri dish test was conducted without Fg (Figure 3). Compared to the water treatment (leaf length: 91.9 mm), the wheat leaves, inoculated with *Nocardioides* sp. SS1 (leaf length: 109.2 mm) and *Marmoricola* sp. MIM116 (leaf length: 107.7 mm), were significantly longer (*P* < 0.05). However, Other DDB treatments did not show a significant difference compared to water-treated controls. Additionally, the leaf lengths inoculated with Fg and those without Fg were not correlated (R^2^ = 0.11, *P* < 0.05).

### 2.4. Nocardioides spp. SS3 and SS4 Reduce the Effects of DON

**Figure 4:**
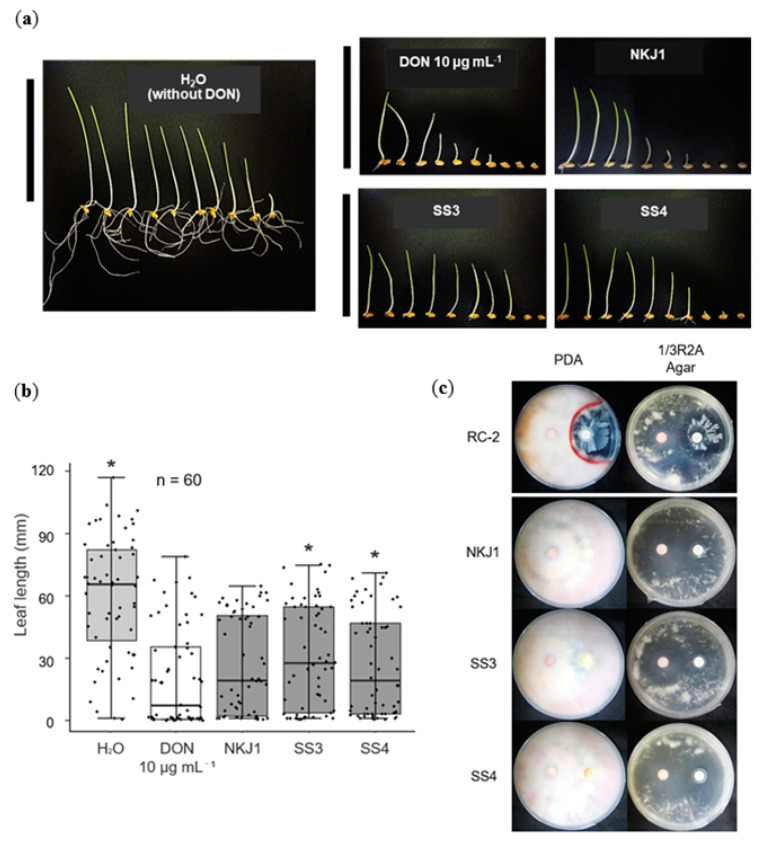
Effects of selected DDBs inoculation on growth of leaf lengths of germinated wheat seed treated with DON (a and b), as well as on *F. graminearum* s. str. 0407011 (Fg) hyphae growth in solid media (c). (**a**) Geminated seeds were treated with DON and incubated with selected DDB strains for 6 days at 28 °C. H_2_O (sterile distilled water) indicates a treatment without DON. (a) The picture of 10 representative in vitro germinated seeds. Left- and right-most seedlings show the longest and shortest leaves, respectively. Black bars indicate 100 mm. (**b**) Box plots showing the lengths of wheat leaves in each petri dish. Medians (solid lines) are shown in each box. The bottom and top of the box indicate the 25th and 75th percentiles, respectively. The bottom and top of the error bars show the 10th and 90th percentiles, respectively. The small dots represent the leaf lengths of each sample. The procedure was conducted 60 times. The leaf lengths, stated with asterisks (*), were significantly different (Mann–Whitney test, *P* < 0.05) to DON 10 µg mL^−1^ treatment. (**c**) Dual culture test of Fg (left filter paper disk) and selected DDBs (right filter paper disk) on solid agar plates. *B. amyloliquefaciens* RC-2 was used as a positive control. On the PDA and 1/3R2A agar plates, the Fg hyphae growth was inhibited by the strain RC-2, and a red pigment was formed around the border of the inhibition zone.

To determine the effects of DON on wheat, as well as its degradation by DDBs, germinated seeds were treated with 10 µg mL^−1^ DON solution (Figure 4a,b). *Devosia* sp. strain NKJ1 and *Nocardioides* strains SS3 and SS4 were chosen for this experiment since these are strains that had demonstrated significant suppression of Fg disease progression in the petri dish test (Figure 2). In wheat seeds treated with DON alone, the leaf lengths (leaf length: 21 mm) were shorter than those of H_2_O treated samples (leaf length: 60.7 mm) (*P* < 0.05), indicating that leaf growth was suppressed by DON (Figure 4a,b). The phytotoxic effect of DON on leaf growth was partially mitigated by *Nocardioides* spp. SS3 (leaf length: 30.9 mm) and SS4 (leaf length: 27 mm) inoculations, and the leaves of these samples were significantly longer than when DON was treated by itself (*P* < 0.05).

### 2.5. None of the DDBs Showed Antagonistic Activity

In order to verify whether the DDBs have a direct inhibitory effect on Fg, their antifungal activity was tested on both PDA and 1/3R2A agar media plates (Figure 4c, only the selected strains were shown). Except for the positive control *B. amyloliquefaciens* RC-2 [39], no inhibition of Fg was detected for any of the DDBs tested. This result indicates that the DDBs tested do not possess antimicrobial compound(s) production ability or other means to affect Fg directly.

## 3. Discussion

Although many DDBs have been isolated and analyzed so far [26,27], little is known about the effect these bacteria have on the FHB pathogen and DON accumulation in wheat plants. Here we found, by using a modified in vitro assay system using wheat seedlings, named the petri dish test, that several DDBs can suppress both the severity of FHB as well as DON accumulation.

Using this petri dish test, we found that wheat leaf lengths were significantly longer in inoculations of Fg together with *Devosia* sp. strain NKJ1 or *Nocardioides* spp. strains SS3 and SS4 compared to the Fg inoculations without DDBs (Figure 2). Previous articles have reported that the leaf length reduction by Fg in the petri dish tests is linked to the pathogenicity of the fungus [33,40]. Our experimental data support the notion that DDBs may be used to restrain the onset of Fg in wheat seeds/seedlings. From the results of the dual culture test on agar plates, it could be concluded that DDBs had no direct antifungal activity on Fg (Figure 4c) and that therefore these DDBs had an indirect MOA against the infection spread of *F. graminearum* in the wheat plants. The results also showed that the petri dish test is a useful tool for the screening of BCA candidates, which act via an indirect MOA.

In our experiments, we could show that DON accumulation in wheat leaves inoculated with both Fg and DDB strains NKJ1, SS3, and SS4 was reduced compared to samples that were only inoculated with Fg (Figure 2c). Furthermore, we could show that the leaf length was highly correlated to DON accumulation (Figure 2c). In the case of DON treatment in the absence of Fg, the leaf length was longer for samples inoculated with the DDB strains SS3 and SS4 than in samples treated with DON alone (Figure 4b). These results indicate that DDBs contribute to the repression of FHB via the degradation of the DON.

We measured DON concentration in wheat samples using a simple HPLC–UV method as described in the Materials and Methods section. Although the sensitivity of this method was low (quantification limit, 2 μg g^−1^) compared to those used in actual crop production processes, this method was useful and sensitive enough for the first screening of DDBs to be deemed promising for suppressing FHB, as the DON concentrations in wheat samples affected by Fg were by far higher in our experiment (Figure 2c).

Of the 14 DDBs tested, 11 strains showed no significant suppression effect against FHB (Figure 2). Especially strain KSM1, isolated from lake water [36], showed the lowest FHB suppression ability. This result suggests that the strain KSM1 either could not colonize on the wheat plants or did not express the genes required for DON-degradation. The reason for this may lie in the test conditions, which did not resemble the conditions present in the natural habitat of this strain. Weller [41] mentioned that for the development of biocontrol agents based on microbial colonization and propagation principles, a selection of microbes residing on the target plant or the rhizosphere thereof is preferable. It is, therefore, reasonable to assume that the strains SS3, SS4, and NKJ1 showed a higher FHB suppression because they were isolated from soil [35] and thereby were better adapted to the environment. On the other hand, it should be noted that even strains that had been isolated from soil and wheat plants were often unable to significantly suppress FHB, indicating that these strains were also unable to degrade DON to the best of their ability. It has previously been reported that the level of DON degradation activity depends not only on the strain, but also on various artificial pre-culture conditions [35], and it is, therefore, reasonable to assume that unfavorable growth conditions are a major limiting factor for successful DON degradation by DDBs on wheat.

Whether DON-degradation is the only MOA in these DDBs, or whether suppression of Fg can occur via other mechanisms as well, is at this point not known. There are reports of BCAs that possess the ability to promote plant growth (plant growth-promoting rhizobacteria; PGPRs) and systemic acquired resistance (SAR, by resistance-inducing beneficial microbes) [42,43]. We found that the strains SS1 and MIM116 possess the ability of plant growth-promotion (Figure 3), indicating that this may be a further MOA. However, the strains showed only little FHB suppression, and it, therefore, seems that the plant growth-promotion was not sufficiently strong to counter the adverse effects of FHB. Cases where SAR is induced via expression of resistance genes by PGPR, including the Actinobacteria *Streptomyces*, are well known [42,43,44,45], implying that the strains SS3 and SS4 in this taxonomic class may have similar potential. Further investigations are therefore needed to examine the ability of SAR induction by these DDBs.

In this study, we established the petri dish test to screen for promising microorganisms that can restrain FHB, as well as DON accumulation, by a simple measurement of the wheat leaf length. This analytical system hence provides useful primary information towards establishing virulence factor/mycotoxins-degrading microorganism treatments for practical use, although further characterization of BCA candidates in pot tests using wheat heads and field experiments is required. This study also provides fresh insights into the potential use of DDBs during periods where the application of chemical fungicides is prohibited, such as pre- and post-harvest.

## 4. Materials and Methods

### 4.1. Chemicals, Strains, and Media

DON was prepared as previously described [35,46]. For culturing of DDBs (Table 1), the media used in this study were three-fold diluted R2A (1/3R2A; Wako, Osaka, Japan) and three-fold diluted Luria–Bertani (1/3LB; Difco, MD, USA). Solid media were prepared by gellan gum powder (plant tissue grade; Wako, Osaka, Japan) (1.6% (w/v)) or agar powder (medium grade; Wako) (1.5% (w/v)). CaCl_2_ solution (containing 11.7 g L^−1^) was added to a final concentration of 2 mM just after autoclaving to solidify the media with gellan gum. *F. graminearum* s. str. 0407011 (Fg) [38] was grown on potato dextrose agar (PDA; Difco).

### 4.2. Fg Microconidia Preparation

Fg mycelia grown on PDA were used to inoculate a 200 mL flask containing 100 mL mung bean broth [47] and shaken on a rotary shaker at 120 rpm and room temperature for one week. The culture was filtered through a 200 mesh filter cloth to remove mycelium and put into a 50 mL tube. The conidial suspension was centrifuged at 2500 × g for 15 min, washed with sterile distilled water (SDW), and adjusted to 1.0 × 10^5^ conidia mL^−1^ for petri dish test and 1.0 × 10^7^ conidia mL^−1^ for antagonistic activity assay, respectively, with a solution containing 0.01% (v/v) Tween 20 as a surfactant.

### 4.3. DON-Degrading Bacteria Suspension

Table 1 shows the DDBs inoculated to wheat seeds. *Nocardioides* spp. strains WSN05-2, SS3, YMN1, SS1, YUL1, PFS1, LS1, and SS4 and *Devosia* spp. strains NKJ1 and NKK1 were cultured on 1/3R2A agar media; *Youhaiella* spp. strains SS5 and RS1, formerly classified as genus *Devosia* [35], were cultured on 1/3LB agar media; *Marmoricola* sp. strain MIM116 and *Sphingomonas* sp. strain KSM1 were cultured on 1/3R2A gellan gum media. All bacteria were cultured at 28 °C in the dark for 7 days. After incubation, bacterial cells were scraped from solid media and suspended in SDW. The bacterial suspension, used for the petri dish test and antagonistic activity assay, was adjusted to OD_600_ of 0.1 and 0.3, respectively.

### 4.4. Wheat Seeds and Petri Dish Test

This biological assay was modified from the in vitro test of Purahong et al. [33] (Figure 1). First, surfaces of cv. Norin No. 61 wheat seeds were sterilized in 2% (v/v) sodium hypochlorite solution for 8 min and washed several times with SDW. Seeds were plated onto two sheets of pre-wetted filter paper (9 cm in diameter; Toyo Roshi, Tokyo, Japan) with 10 mL SDW in a petri dish (9 cm in diameter). Wheat seeds were incubated at 4 °C for 5 days in the dark, followed by further incubation in the dark at 20 °C until germination.

Secondly, each 1 mL bacterial suspension (OD_600_ of 0.1) was inoculated onto the germinated wheat seeds in each petri dish and put in the dark at 20 °C for 1 day. Ten germinated seeds were then randomly selected and put on two sheets of filter paper (5.5 cm in diameter; Toyo Roshi) in another petri dish (6 cm in diameter) with the germinated seeds pointing upwards. Then 5 mL of SDW or Fg conidia suspension was then dropped on them. The petri dishes were incubated in a plastic container and kept at 20 °C for 6 days in the dark.

Thirdly, 6 days after inoculation (DAI), the length of the leaves and the lesions were measured carefully, and disease severity was calculated using ratio of lesion length to whole leaf length. Afterward, the wheat samples were stored at −30 °C until DON accumulation was measured.

The petri dish test was also conducted with 10 µg mL^−1^ DON solution containing 0.01% (v/v) Tween 20, without Fg. After bacterial inoculation on germinated seeds, 5 mL SDW or DON 10 µg mL^−1^ solution were dropped into a petri dish containing two sheets of filter paper; then 10 wheat seeds were laid on top. At 6 DAI, the lengths of wheat leaves and the lengths of the lesions were measured.

### 4.5. DON Extraction and HPLC Analysis

The tested wheat samples were stored at −30 °C in a refrigerator until use, and DON extraction was conducted as described [48] with slight modification. The total volume of 200 µL extraction solvent (CH_3_CN:H_2_O:HAc, 79:20:1 (v/v/v)) was added to 50 mg (dry weight) ground wheat sample. The samples were extracted using a MicroMixer E 36 rotary shaker (TAITEC, Aichi, Japan) at room temperature for 90 min and subsequently fractionated by centrifugation at 20 °C, 13,000 × g for 2 min. Then 30 µl of the supernatant was transferred to a new 1.5 mL plastic tube and diluted with the same amount of extraction solvent (CH_3_CN:H_2_O:HAc, 79:20:1 (v/v/v)). Then 10 µL of the extract were injected into a high liquid performance chromatography (HPLC) system after filtering through a 0.45-µm membrane (Millipore, Darmstadt, Germany). The HPLC system (Shimadzu, Kyoto, Japan) consisted of an LC-20AD pump, a SPD-M20A absorbance detector, a Develosil ODS-UG-3 (4.6 mm ID × 150 mm; Nomura, Aichi, Japan) column, and LC solution software version 1.25 SP5 (Shimadzu). The mobile phase contained acetonitrile and water (15:85 (v/v)) at a flow rate of 1.0 mL min^−1^. The detection wavelength was set at 220 nm. The column temperature was 40 °C. Authentic DON was detected at a retention time of around 4.1 min. Detection and quantification limits for DON were 2 μg g^−1^ with a signal-to-noise ratio of 5:1. Recoveries from wheat leaf samples spiked with 2 μg g^−1^, 10 μg g^−1^, and 50 μg g^−1^ were 72.6%, 80.2%, and 90.5%, respectively.

### 4.6. Antagonistic Activities Assay against F. graminearum on Solid Media

A dual-culture plate test was conducted to evaluate the antibiotic activity of DDBs against Fg. An iturin-producing *B. amyloliquefaciens* strain RC-2 [38], grown on PDA and 1/3R2A agar plates, was used as a positive control. The conidia and bacterial suspensions were prepared, as already described. Two paper disks (8 mm in diameter; Toyo Roshi) were put on PDA and 1/3R2A agar media, and 50 µL of each of the two suspensions was pipetted onto the paper disks. The plates were incubated at 28 °C for 4 days in the dark, and the antifungal activities of DDBs were determined by detection of the pathogen’s hyphal growth inhibition zone.

### 4.7. Statistical Analysis

Statistical analysis of the data was carried out using R (version 3.4.0; the Comprehensive R Active Network) [49]. Significant differences between the means were determined by using an unpaired Student’s *t*-test (*P* < 0.05), unpaired Welch’s *t*-test (*P* < 0.05), Mann–Whitney test (*P* < 0.05), and ANOVA incorporating Tukey–Kramer test variance analysis (*P* < 0.05). We analyzed the relationship between the data by Pearson product–moment correlation coefficient (Pearson’s *r*).

## Figures and Tables

**Table 1:** The DON-degrading bacteria (DDBs) used in this study.

Class	Strain [Ref. No.]	Source of Isolation	Closely Related Species (% 16S rRNA Gene Sequence Similarity)
Actinobacteria	WSN05-2 [34]	Wheat field soil	*Nocardioides panacihumi* Gsoil 616 (98.37)
	SS3 [35]	Wheat field soil	*Nocardioides panacihumi* Gsoil 616 (98.47)
	YMN1 [35]	Uncultivated soil	*Nocardioides panacihumi* Gsoil 616 (98.33)
	SS1 [35]	Wheat field soil	*Nocardioides panacihumi* Gsoil 616 (98.98)
	YUL1 [35]	Wheat field soil	*Nocardioides panacihumi* Gsoil 616 (98.73)
	PFS1 [35]	Paddy field soil	*Nocardioides panacihumi* Gsoil 616 (98.72)
	SS4 [35]	Wheat field soil	*Nocardioides ginsengisegetis* Gsoil 485 (100)
	LS1 [35]	Wheat leaf	*Nocardioides ginsengisegetis* Gsoil 485 (100)
	MIM116 [36]	Wheat head	*Marmoricola aequoreus* SST-45 (98.80)
Alphaproteobacteria	SS5 [35] †	Wheat field soil	*Youhaiella tibetensis* fig4 (99.84)
	RS1 [35] †	Wheat field soil	*Youhaiella tibetensis* fig4 (99.10)
	NKJ1 [35]	Paddy field soil	*Devosia insulae* DS-56 (99.34)
	NKK1 [35]	Wheat field soil	*Devosia insulae* DS-56 (99.10)
	KSM1 [37]	Lake water	*Sphingomonas naphthae* DKC-5-1 (97.40)

^†^ Strain SS5 and RS1 were formerly classified as genus *Devosia*.
